# A two-miRNA signature (miR-33a-5p and miR-128-3p) in whole blood as potential biomarker for early diagnosis of lung cancer

**DOI:** 10.1038/s41598-018-35139-3

**Published:** 2018-11-12

**Authors:** Jinchang Pan, Chengwei Zhou, Xiaodong Zhao, Jinxian He, Hui Tian, Weiyu Shen, Ying Han, Jun Chen, Shuai Fang, Xiaodan Meng, Xiaofeng Jin, Zhaohui Gong

**Affiliations:** 1 Department of Biochemistry and Molecular Biology, Medical School of Ningbo University, Ningbo, 315211 China; 2 Zhejiang Provincial Key Laboratory of Pathophysiology, Medical School of Ningbo University, Ningbo, 315211 China; 3 Department of Thoracic Surgery, The Affiliated Hospital of Medical School of Ningbo University, Ningbo, 315020 China; 4 Department of Thoracic Surgery, The Affiliated Ningbo Medical Center Lihuili Eastern Hospital of Medical School of Ningbo University, Ningbo, 315048 China; 5 Department of Radiation Oncology, The Affiliated Yinzhou Renmin Hospital of Medical School of Ningbo University, Ningbo, 315040 China

## Abstract

MicroRNAs (MiRNAs) have been found to be dysregulated in lung cancer tissues compared to their matched paracancerous tissues. However, the roles of miRNAs in peripheral blood as potential biomarkers for early diagnosis of lung cancer remain poorly understood. Here we found that miR-33a-5p and miR-128-3p were down-regulated in lung cancer tissues and cell lines. The expression levels of miR-33a-5p and miR-128-3p in lung cancer tissues were significantly correlated to TNM stages. MiR-128-3p in lung cancer tissues was also remarkably related to smoking and tumor size. The relative expression levels of miR-33a-5p and miR-128-3p were positively correlated in lung cancer tissues. Notably, miR-33a-5p and miR-128-3p in whole blood of lung cancer patients or early-stage lung cancer patients (TNM stage I-II) were lowly expressed as compared with that in healthy controls. The receiver operating characteristic curve (ROC) analyses revealed higher area under the ROC curve (AUC) values and higher sensitivity/specificity of miR-33a-5p and miR-128-3p alone and in combination were superior to that of traditional tumor markers (CYFR21-1, NSE and CA72-4). Importantly, both miR-33a-5p and miR-128-3p in whole blood were highly stable even under different harsh conditions. The results demonstrate that tumor suppressor miR-33a-5p/miR-128-3p in whole blood can serve as novel biomarkers for the early detection of lung cancer.

## Introduction

Lung cancer can be divided into non-small cell lung cancer (NSCLC) and small cell lung cancer (SCLC) according to different pathological types, and NSCLC accounts for 80–85% of all lung cancers^1^. Although the current diagnosis and treatment for lung cancer have made great progress in past decades, the mortality rate in lung and bronchus cancers is 26% and 25%, respectively, and the 5-year survival rate of lung cancer is only 5%^2^. Therefore, early diagnosis for lung cancer is particularly important.

MicroRNAs (miRNAs) are small, conserved, non-coding RNA molecules of approximately 19-25 nucleotides in size that bind to the 3′-untranslated region of a target mRNA resulting in mRNA degradation and/or translation repression^3,4^. Studies have reported that miRNAs can be involved in lung cancer cell proliferation, migration, invasion and drug resistance^5–9^. The miRNAs present in the blood are usually called circulating miRNAs^10^. Circulating miRNAs can be stably present in the blood in the form of Ago2-miRNA protein complexes to protect against RNase degradation and multiple freeze-thaw cycles^11^. Therefore, the differentially expressed circulating miRNAs in lung cancer patients and healthy humans can be used as novel biomarkers for diagnosis of lung cancer^12^. MiR-33a-5p is an intronic miRNA located in intronic sequences of the sterol-response-element-binding protein gene 2 (SREBP2), it has been reported that miR-33a-5p acts as a tumor suppressor and inhibits cancer cell proliferation and aggressiveness in lung cancer^13–15^. In addition, miR-128 is significantly down-regulated in NSCLC tissues and cancer cells, *in vivo* restoration of miR-128 significantly suppresses tumourigenicity of A549 cells in nude mice and inhibits both angiogenesis and lymphangiogenesis of tumor xenografts^16^. Importantly, antagonism of miR-128-3p potently reverses metastasis and chemoresistance of highly malignant NSCLC cells, which could be completely reversed by restoring Wnt/β-catenin and TGF-β activities, suggesting that miR-128-3p might be a potential target against both metastasis and chemoresistance in NSCLC^17^.

In our previous studies, we have found that two miRNAs (miR-33a-5p and miR-128-3p) are down-regulated in lung cancer tissues compared to paired normal tissues^18,19^. However, the clinical significance of these two miRNAs in early diagnosis of lung cancer remains unclear. Compared with tissue samples, blood specimens are easier to collect and miRNA in whole blood can serve as a new type of non-invasive biomarker in the diagnosis of lung cancer. In present study, we detected the expression levels of miR-33a-5p and miR-128-3p in whole blood samples to evaluate the values of these two miRNAs as biomarkers for early diagnosis of lung cancer.

## Results

### MiR-33a-5p and miR-128-3p were significantly down-regulated in lung cancer tissues and lung cancer cell lines

**Figure 1:**
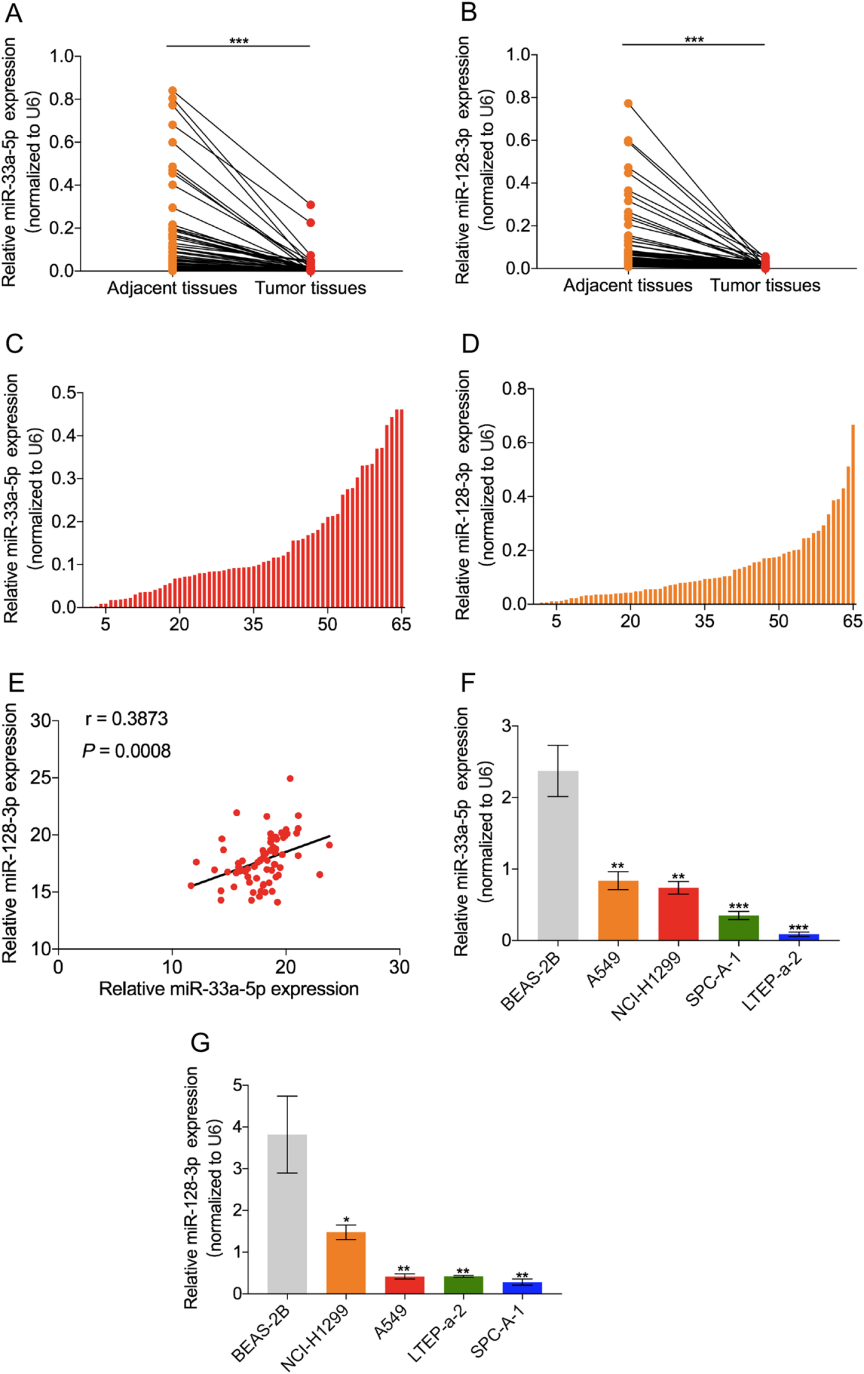
Both miR-33a-5p and miR-128-3p were down-regulated in lung cancer tissues and lung cancer cell lines. MiR-33a-5p (**A**) and miR-128-3p (**B**) showed a statistically down-regulated expression in each lung cancer tissues and the matched adjacent normal tissues (n = 65, *P* < 0.001). The relative expression of miR-33a-5p (**C**) and miR-128-3p (**D**) in 65 paired lung cancer tissues and matched adjacent normal tissues. Results were presented as the fold-change in tumor tissues compared to adjacent normal tissues. (**E**) Pearson correlation coefficient showed that the relative expressions of miR-33a-5p and miR-128-3p were significantly positively correlated in lung cancer tissues (*P* = 0.0008). Compared with normal lung epithelial cell (BEAS-2B), the relative expressions of miR-33a-5p (**F**) and miR-128-3p (**G**) were significantly lower in four lung cancer cell lines (NCI-H1299, A549, SPC-A-1, and LTEP-a-2, *P* < 0.05). *P < 0.05, **P < 0.01, ***P < 0.001.

To confirm the expression levels of miR-33a-5p and miR-128-3p in lung cancer patients, we detected their expression levels in 65 lung cancer tissues and the paired normal adjacent tissues using qRT-PCR. As was expected, the melting curve showed that the amplified product of miR-33a-5p/miR-128-3p generated a single peak (Fig. S1A,B). The qRT-PCR results showed that miR-33a-5p and miR-128-3p were significantly down-regulated in lung cancer tissues compared to adjacent normal tissues (*P* < 0.001, Fig. 1A,B). Notably, in each pair of cancer and adjacent normal tissue, the fold change of miR-33a-5p was 0.001~0.46 (Fig. 1C), while the fold change of miR-128-3p was 0.001~0.67 (Fig. 1D). Interestingly, the relative expression levels of miR-33a-5p and miR-128-3p were positively correlated in lung cancer tissues (*P* < 0.001, Fig. 1E). In addition, we further validated the expression levels of miR-33a-5p and miR-128-3p in lung cancer cell lines. Compared with human normal lung epithelial cells (BEAS-2B), both miR-33a-5p of and miR-128-3p were lowly expressed in lung cancer cell lines (NCI-H1299, A549, SPC-A-1 and LTEP-a-2) (*P* < 0.05, Fig. 1F,G). Together, these two tumor suppressor miRNAs were remarkably down-regulated in lung cancer.

### MiR-33a-5p was closely correlated to TNM stage, while miR-128-3p was strongly related to smoking, tumor size and TNM stage in lung cancer patients

To investigate the relationship between miR-33a-5p/miR-128-3p expression levels and clinicopathological factors in lung cancer patients, we collected and analyzed the clinical data of 65 proven cases. Based on the constitutive expression levels of miR-33a-5p/miR-128-3p, 65 cases were divided into high expression group (n = 33) and low expression group (n = 32) by the median. We did not find a significant association of miR-33a-5p expression with patients’ sex (*P* = 0.6942), age (*P* = 0.3781), smoking (*P* = 0.2669), pathology classification (*P* = 0.8191), tumor location (*P* = 0.8753), differentiated degree (*P* = 0.5902) and tumor size (*P* = 0.8208). However, the expression of miR-33a-5p was significantly correlated with tumor node metastasis (TNM) stage (*P* = 0.0133) (Table 1). Similarly, we also found there was no significant association of miR-128-3p expression with patients’ sex (*P* = 0.7083), age (*P* = 0.3843), pathology classification (*P* = 0.1535), tumor location (*P* = 0.6854) and differentiated degree (*P* = 0.11) (Table 2). Whereas miR-128-3p expression was strongly related to tumor size (*P* = 0.0025) and TNM stage (*P* = 0.0076). Interestingly, miR-128-3p was also found to be correlated with smoking (*P* = 0.0178) (Table 2). In general, the expression of miR-33a-5p/miR-128-3p were closely correlated with tumor growth in the early stage.

**Table 1:** The relationships between the expression levels of miR-33a-5p(2^−ΔCt^) in lung cancer tissues and the clinicopathological factors of patients with lung cancer.

Characteristics	Case No.	MiR-33a-5p relative level (2^−ΔCt^)		χ^2^ test	*P* value
		High	Low		
Total cases	65	33	32		
Gender					
Male	37	18	19	0.1545	0.6942
Female	28	15	13		
Age (years)					
≤60	21	9	12	0.777	0.3781
>60	44	24	20		
Smoking history					
Yes	30	13	17	1.233	0.2669
No	35	20	15		
Histology					
Adenocarcinoma	45	22	23	0.3991	0.8191
Squamous carcinoma	17	9	8		
Other type	3	2	1		
Tumor location					
Left	25	13	12	0.02462	0.8753
Right	40	20	20		
Differentiation					
High and moderate	14	8	6	0.29	0.5902
Poor	51	25	26		
Tumor size(cm)					
≤3	50	25	25	0.05129	0.8208
>3	15	8	7		
TNM stage					
I–II	41	25	16	6.128	0.0133^*^
III–IV	24	7	17		

^*^P < 0.05.

**Table 2:** The relationships between the expression levels of miR-128-3p(2^−ΔCt^) in lung cancer tissues and the clinicopathological factors of patients with lung cancer.

Characteristics	Case No.	MiR-128-3p relative level (2^−ΔCt^)		χ^2^ test	*P* value
		High	Low		
Total cases	65	33	32		
Gender					
Male	33	16	17	0.14	0.7083
Female	32	17	15		
Age (years)					
≤60	23	10	13	0.7571	0.3843
>60	42	23	19		
Smoking history					
Yes	27	9	18	5.618	0.0178^*^
No	38	24	14		
Histology					
Adenocarcinoma	47	22	25	3.748	0.1535
Squamous carcinoma	14	10	4		
Other type	4	1	3		
Tumor location					
Left	26	14	12	0.1641	0.6854
Right	39	19	20		
Differentiation					
High and moderate	11	8	3	2.554	0.11
Poor	54	25	29		
Tumor size(cm)					
≤3	51	34	17	9.168	0.0025**
>3	14	3	11		
TNM stage					
I–II	42	29	13	7.116	0.0076**
III–IV	23	8	15		

^*^P < 0.05, **P < 0.01.

### MiR-33a-5p and miR-128-3p had higher diagnostic value in lung cancer tissues

**Figure 2:**
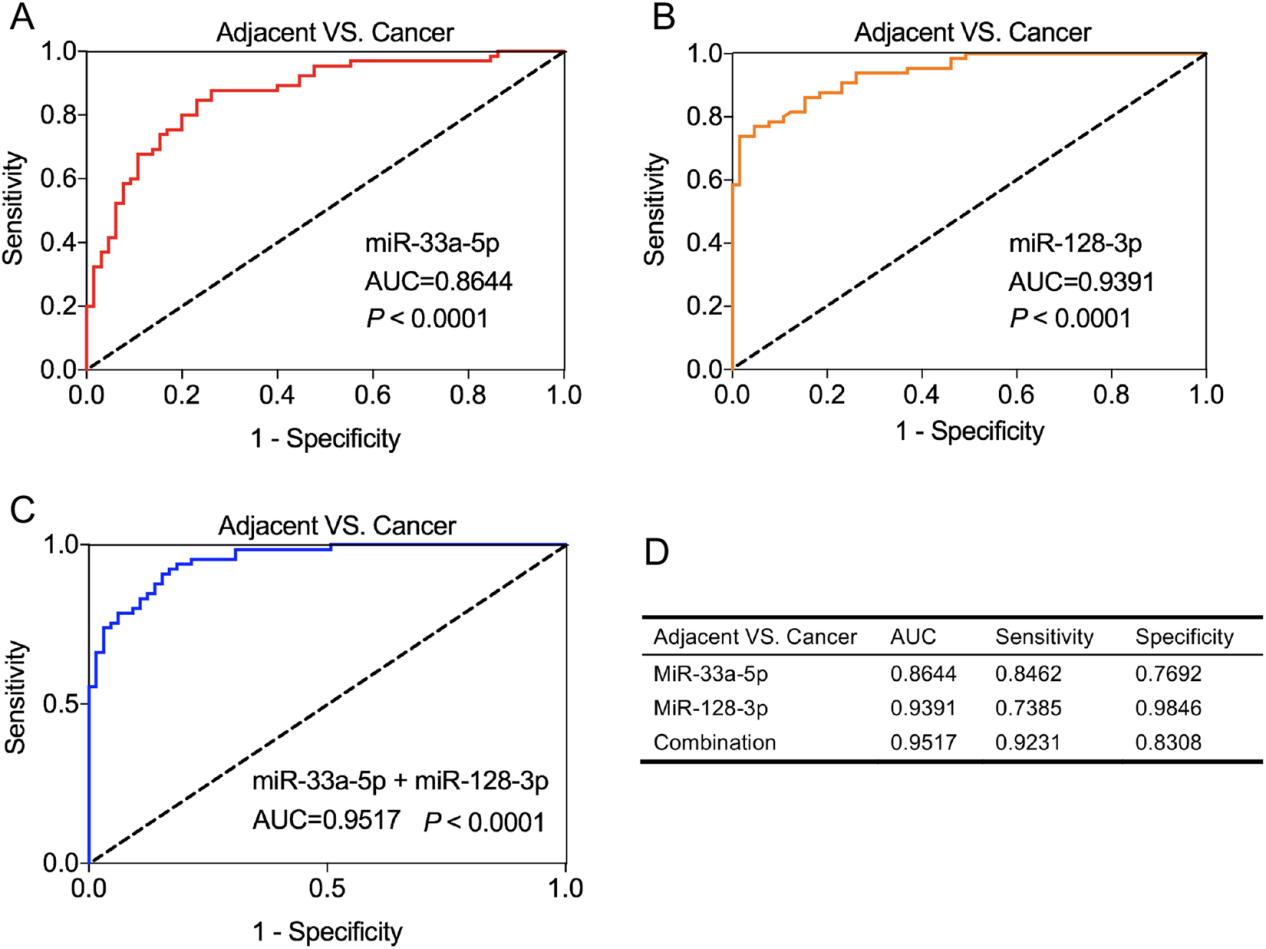
Diagnostic value of miR-33a-5p and miR-128-3p in lung cancer tissues. The ROC analysis for detection of lung cancer tissues and corresponding adjacent normal tissues using miR-33a-5p (**A**), miR-128-3p (**B**), and miR-33a-5p + miR-128-3p (**C**). (**D**) The comparison of diagnostic values between miR-33a-5p, miR-128-3p and the combination.

We further evaluated the diagnostic value of miR-33a-5p and miR-128-3p in lung cancer tissues and corresponding adjacent normal tissues by constructing ROC curves. The ROC analysis showed that the AUC of miR-33a-5p was 0.8644 (95% confidence interval (CI) = 0.8016 to 0.9271, sensitivity = 84.62% and specificity = 76.92%, Fig. 2A,D), and the AUC of miR-128-3p was 0.9391 (95% CI = 0.9022 to 0.9759, sensitivity = 73.85% and specificity = 98.46%, Fig. 2B,D). Moreover, the combination of miR-33a-5p and miR-128-3p yielded a higher AUC value at 0.9517 (95% CI = 0.9199 to 0.9835, sensitivity = 92.31% and specificity = 83.08%, Fig. 2C,D). The results indicated that both miR-33a-5p and miR-128-3p has individual diagnostic value and the combined diagnostic value of these two miRNAs is much higher than that of any single miRNA.

### MiR-33a-5p and miR-128-3p were lowly expressed in whole blood and served as minimally invasive biomarker to diagnose lung cancer

**Figure 3:**
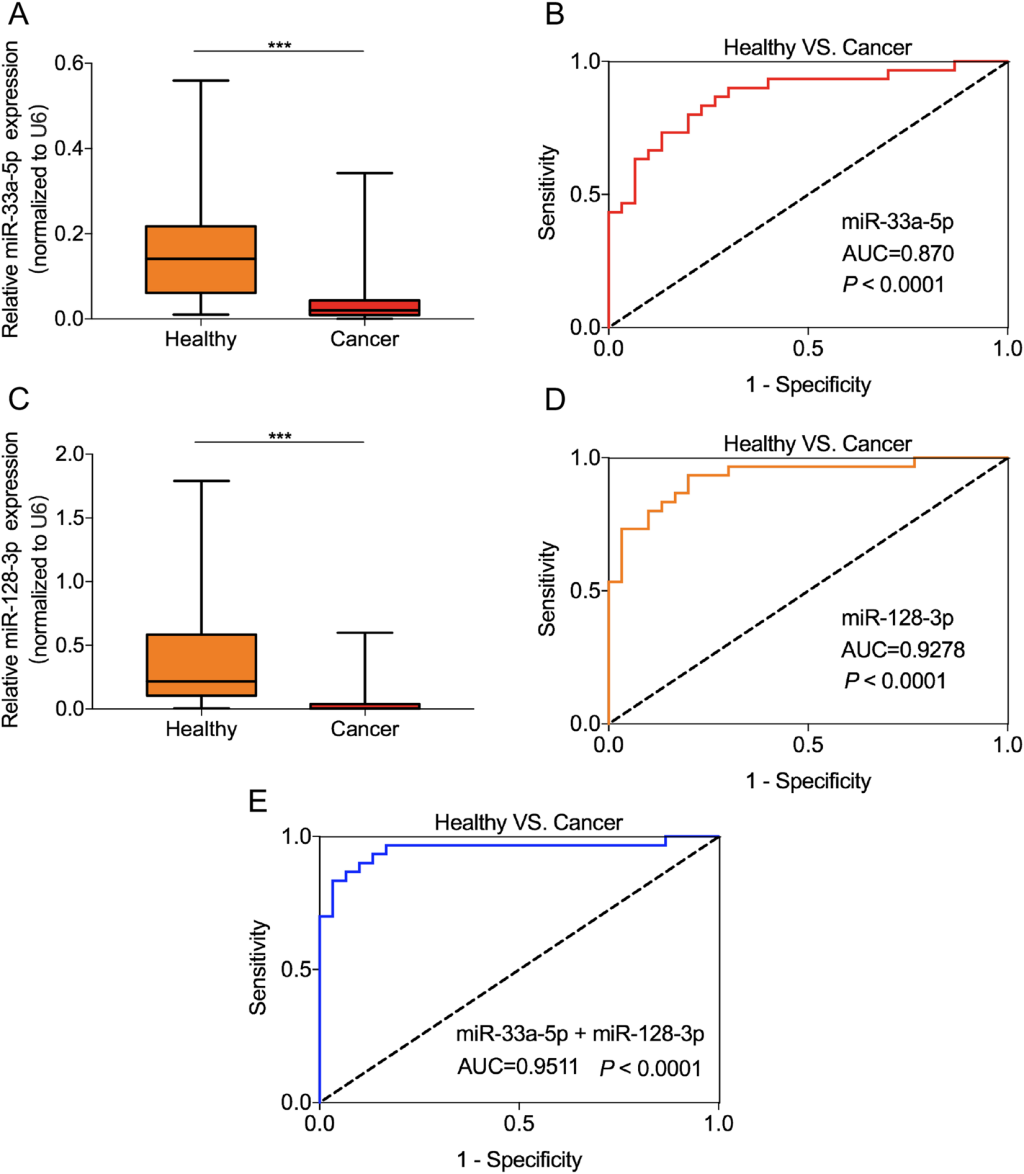
Both miR-33a-5p and miR-128-3p were lower expressed in whole blood with lung cancer and had greater diagnostic value. MiR-33a-5p (**A**) and miR-128-3p (**C**) were down-regulated in whole blood from lung cancer patients compared to that from healthy controls (n = 90, *P* < 0.001). The ROC analysis for detection of lung cancer patients from health controls using miR-33a-5p (**B**), miR-128-3p (**D**), and miR-33a-5p + miR-128-3p (**E**). ***P < 0.001.

To reveal the clinical value of these two miRNAs in non-invasive diagnosis for lung cancer, we analyzed the circulating miR-33a-5p/miR-128-3p in whole blood from 90 patients with lung cancer and 90 healthy controls. The qRT-PCR results showed that miR-33a-5p and miR-128-3p expression levels were significantly down-regulated in whole blood from lung cancer patients compared with that from in healthy controls (*P* < 0.001, Fig. 3A,C). We further evaluated the diagnostic values of miR-33a-5p and miR-128-3p in whole blood with lung cancer and healthy controls. The corresponding AUC value of miR-33a-5p was 0.870 (95% CI = 0.7786 to 0.9614, sensitivity = 86.67% and specificity = 73.33%, Fig. 3B), and the AUC value of miR-128-3p was 0.9278 (95% CI = 0.8616 to 0.9939, sensitivity = 93.33% and specificity = 80%, Fig. 3D). Correspondingly, we used miR-33a-5p and miR-128-3p to create a combined diagnostic model using logistic regression models. The combined diagnostic model showed the AUC of combined two circulating miRNAs was up to 0.9511 (95% CI = 0.8904 to 1.012, sensitivity = 96.67% and specificity = 83.33%, Fig. 3E). These findings indicated that the circulating miR-33a-5p/miR-128-3p were lowly expressed in whole blood of lung cancer patients and had the greater diagnostic value for diagnosis of lung cancer.

### Whole blood miR-33a-5p and miR-128-3p could be used to distinguish early-stage lung cancer patients from healthy controls

**Figure 4:**
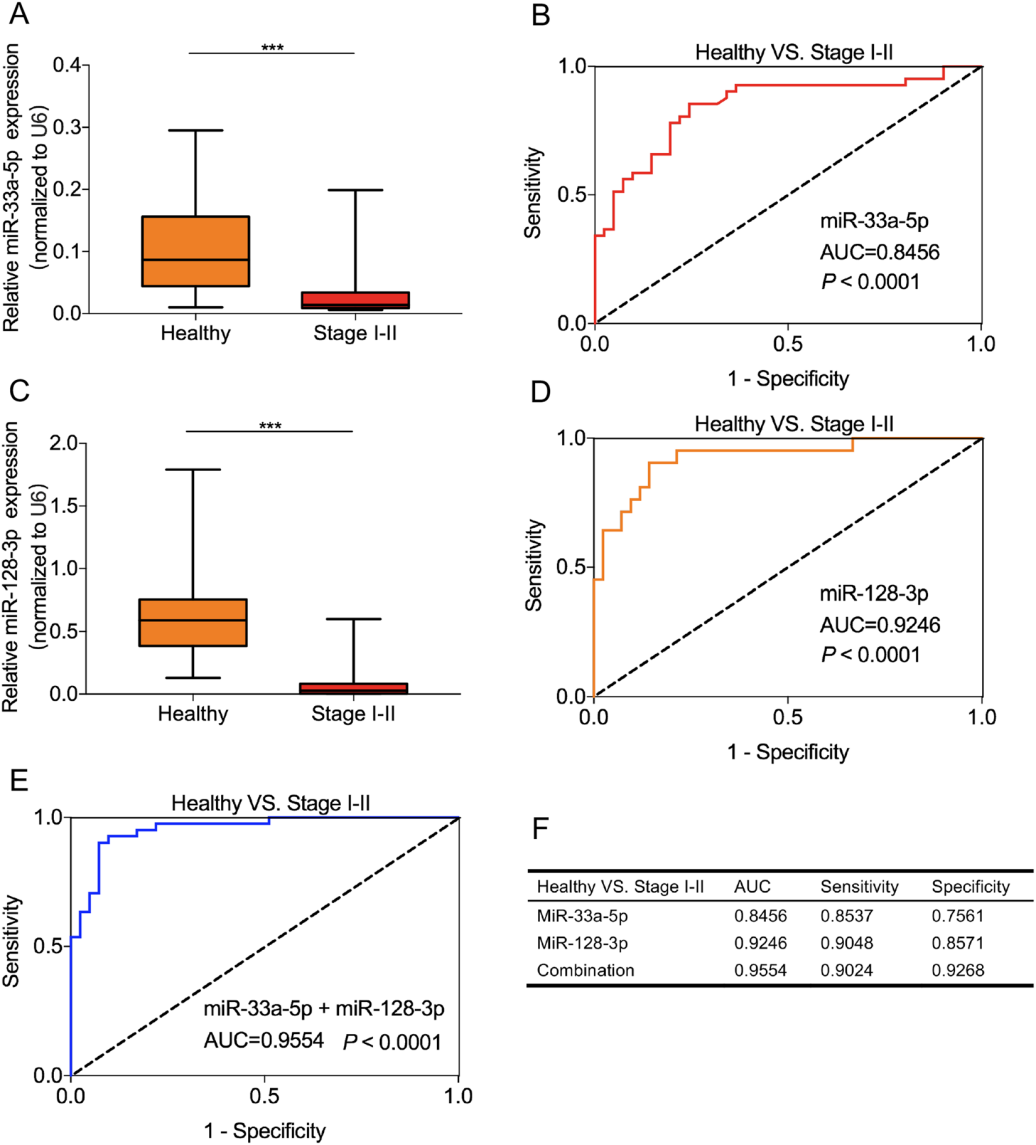
MiR-33a-5p and miR-128-3p could be used to distinguish early-stage lung cancer patients from healthy controls. MiR-33a-5p (**A**) and miR-128-3p (**C**) were down-regulated in early lung cancer patients (TNM stage I-II) compared to healthy controls (*P* < 0.001). The ROC analysis for detection of early lung cancer patients from health controls using miR-33a-5p (**B**), miR-128-3p (**D**), and miR-33a-5p + miR-128-3p (**E**). (**F**) The comparison of diagnostic values between miR-33a-5p, miR-128-3p and the combination. ***P < 0.001.

To further verify the diagnostic value of miR-33a-5p and miR-128-3p in early-stage lung cancer, we detected the relative expression of miR-33a-5p in 41 patients with lung cancer at early stage (TNM stage I-II) and 41 healthy controls, and miR-128-3p in 42 patients with lung cancer at early stage (TNM stage I–II) and 42 healthy controls. The results showed that the expression levels of circulating miR-33a-5p and miR-128-3p in healthy controls were significantly higher than that in early-stage lung cancer patients (*P* < 0.001, Fig. 4A,C). The AUC values of miR-33a-5p and miR-128-3p were 0.8456 (95% confidence interval (CI) = 0.7586 to 0.9327, sensitivity = 85.37% and specificity = 75.61%, Fig. 4B,F) and 0.9246, respectively (95% CI = 0.8677 to 0.9815, sensitivity = 90.48% and specificity = 85.71%, Fig. 4D,F). Interestingly, the combined two-miRNA signature yielded higher values of AUC and specificity (AUC = 0.9554; 95% CI = 0.9144 to 0.9964, sensitivity = 90.24% and specificity = 92.68%, Fig. 4E,F). These results indicated that miR-33a-5p, miR-128-3p, and a two-miRNA signature could be used as novel biomarkers for early diagnosis of lung cancer.

### MiR-33a-5p and miR-128-3p had higher diagnostic value than traditional tumor markers for lung cancer

**Figure 5:**
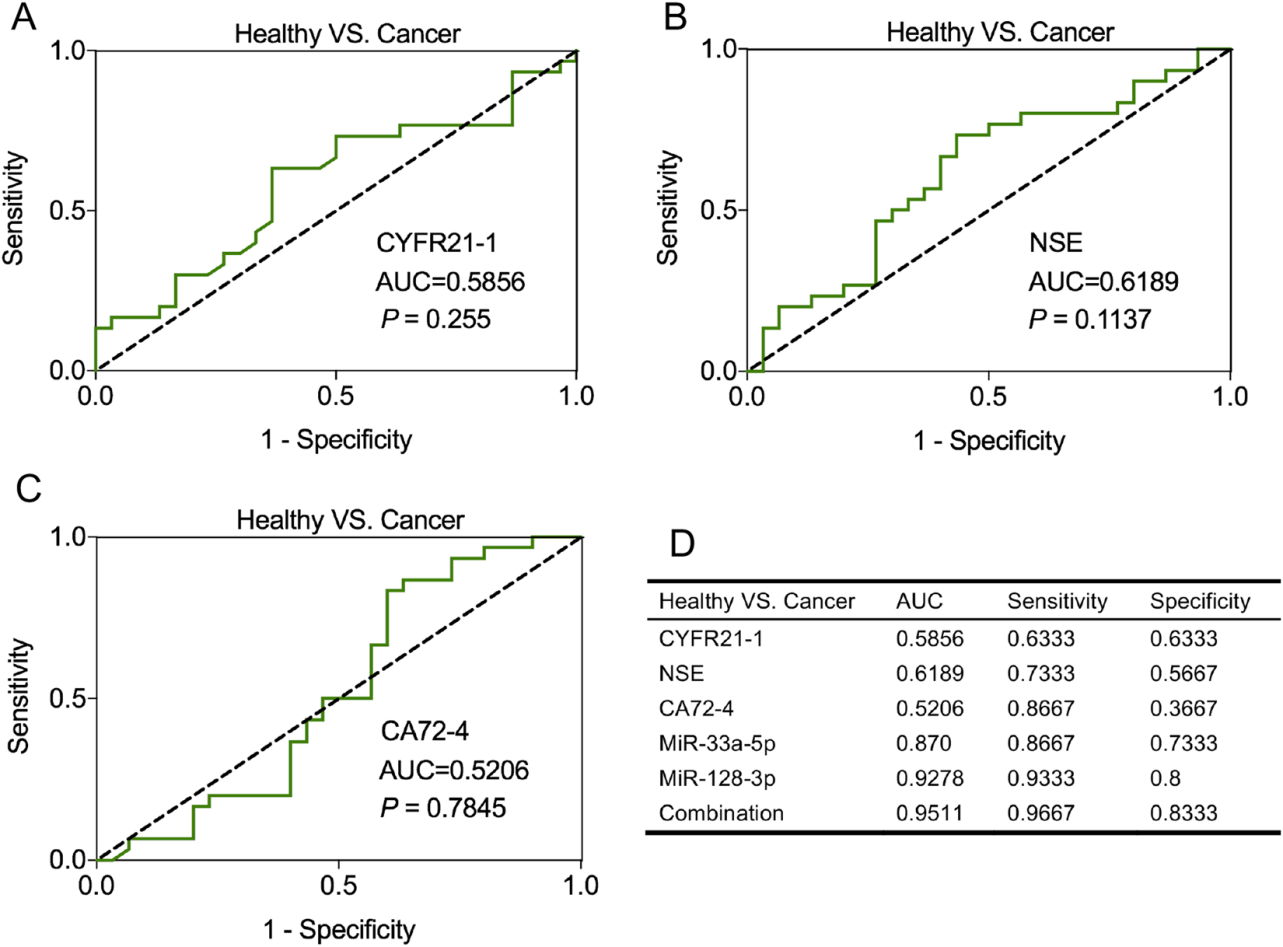
Diagnostic performance of miR-33a-5p/miR-128-3p and three traditional tumor markers for detection of lung cancer. The ROC analysis for detection of lung cancer using traditional tumor markers CYFR21-1 (**A**), NSE (**B**) and CA72-4 (**C**). (**D**) The comparison of diagnosis values between miR-33a-5p/miR-128-3p and the combination and three traditional tumor markers.

We compared the diagnostic values of miR-33a-5p, miR-128-3p and their combination with three commonly used tumor markers (cytokeratin-19-fragment (CYFR21-1), neuron-specific enolase (NSE) and cancer antigen 72-4 (CA72-4)), and obtained the following AUC values: CYFR21-1, 0.5856 (95% CI = 0.4387 to 0.7324, sensitivity = 63.33% and specificity = 63.33%, Fig. 5A,D), NSE, 0.6189 (95% CI = 0.4748 to 0.763, sensitivity = 73.33% and specificity = 56.67%, Fig. 5B,D), CA72-4, 0.5206 (95% CI = 0.3684 to 0.6727, sensitivity = 86.67% and specificity = 36.67%, Fig. 5C,D). In summary, the results showed that the diagnostic values of miR-33a-5p and miR-128-3p were superior to three traditional tumor markers, and the AUC value of the combined diagnosis was not only higher than that of the individual miRNA, but also higher than that of any single traditional biomarker (Fig. 5D), suggesting the highest diagnostic performance.

### MiR-33a-5p and miR-128-3p were stably expressed in whole blood under harsh conditions

**Figure 6:**
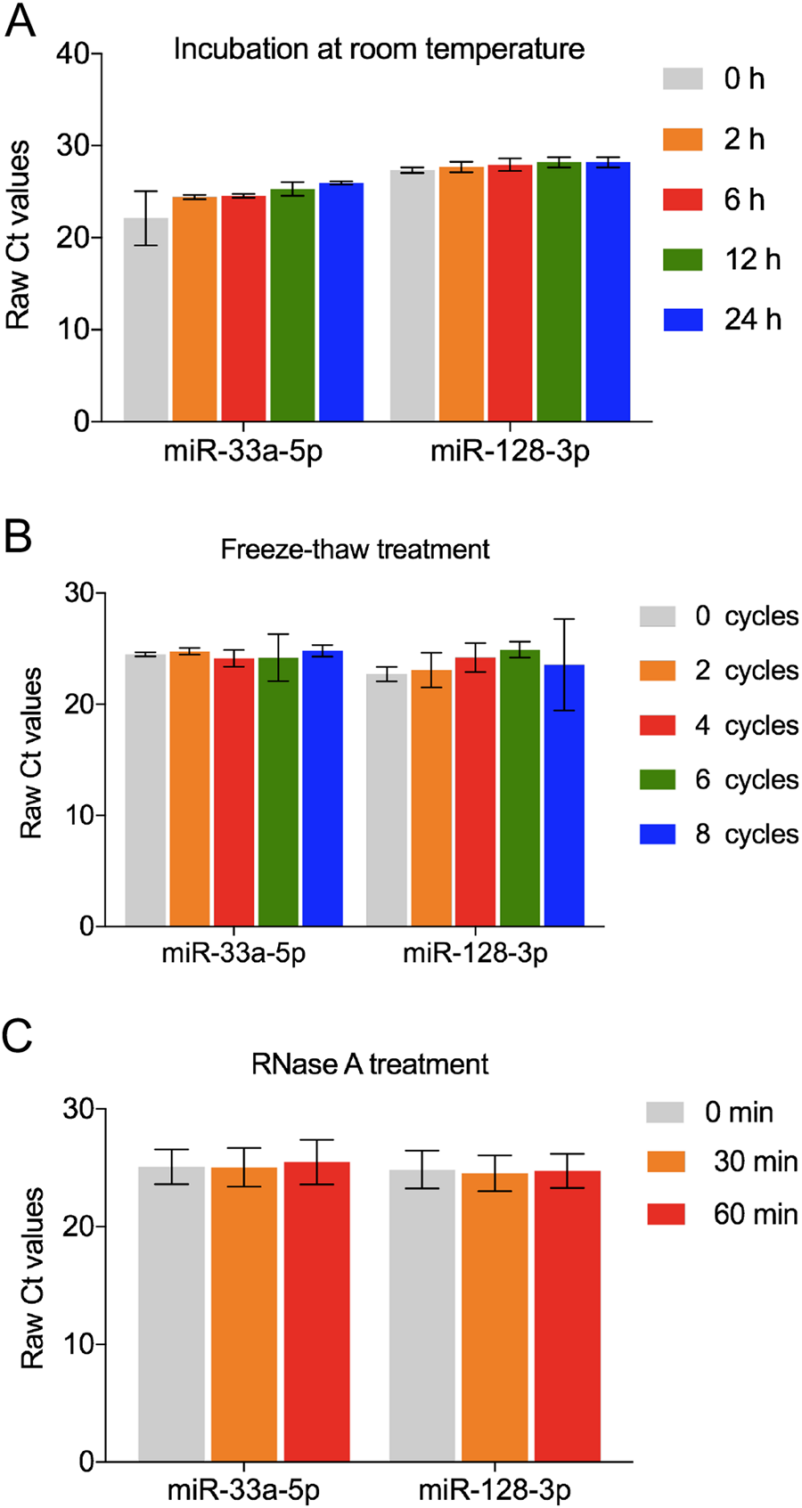
Stability test for miR-33a-5p and miR-168-3p. The fresh whole blood samples from 12 patients with lung cancer were exposed to different harsh conditions: incubating at room temperature for 0, 2, 6, 12, 24 h (**A**), treating with 0, 2, 4, 6, 8 repetitive freeze-thaw cycles (**B**) and RNase A digestion for 0, 30, 60 min at 37 °C (**C**). No significant difference was observed in each group. *P* > 0.05.

To test the stability of miR-33a-5p and miR-128-3p in whole blood of lung cancer patients, we collected fresh whole blood from 12 patients with lung cancer, and divided into 3 groups (4 in each group). The first group was incubated at room temperature for different times (0, 2, 6, 12, 24 h), the second group was repeated for different freeze-thaw cycles (0, 2, 4, 6, 8 cyles), and the third group was treated with RNase A digestion for different times (0, 30, 60 min). The results of qRT-PCR showed that the relative expression levels of miR-33a-5p and miR-128-3p did not significantly change under the different incubation time at room temperature (Fig. 6A). Similarly, no significant difference of the miRNA expression was found between 0 to 8 cycles of freeze-thaw treatment (Fig. 6B). Likewise, the RNase A treatment for different time did not markedly affect the raw Ct values of the two circulating miRNAs (Fig. 6C). These results indicated that both miR-33a-5p and miR-128-3p was stably expressed in whole blood under harsh conditions. Therefore, the stability of these two circulating miRNAs in whole blood demonstrates the potential of being diagnostic biomarkers for lung cancer.

## Discussion

In our previous studies, we found that miR-33a-5p inhibits NSCLC epithelial-mesenchymal transition (EMT) and serves as a prognostic factor for NSCLC^18^. Furthermore, we discovered that miR-128-3p directly targets PFK liver type (PFKL) in lung cancer cells and regulates endogenous expression of PFKL at both mRNA and protein levels. Knockdown of miR-128 has been shown to promote lung cancer cell growth and colony formation^19^. However, the correlation between miR-33a-5p/miR-128-3p and the pathological features of lung cancer patients is still not well known. In current study, we revealed the clinical values of miR-33a-5p and miR-128-3p in peripheral blood for early diagnosis of lung cancer.

In existing studies, miR-33a was found to be decreased in NSCLC tissues compared with the para-carcinoma tissues. Also, miR-33a could inhibit lung cancer cell proliferation, cell cycle progression, and migration by targeting cullin-associated NEDD8-dissociated protein 1^20^. Compared to adjacent non-tumor tissues, miR-128 was down-regulated in lung cancer tissues and miR-128 induced apoptosis in lung cancer cell lines^21^. These results are consistent with our findings, that miR-33a-5p and miR-128-3p were lowly expressed in lung cancer tissues and lung cancer cell lines. In addition, miR-33a-5p and miR-128-3p exhibited lower expression in colorectal cancer^22^, osteosarcoma^23^, breast cancer^24^ and pancreas cancer^25^, suggesting that miR-33a-5p and miR-128-3p might act as tumor suppressors in the progression of cancer and have the potential diagnostic value in cancer.

It is particularly important to improve the early diagnosis and early treatment of patients with lung cancer. MiRNAs have been proven to be targets in cancer therapy and to be biomarkers in cancer diagnosis^11,26,27^. For lung cancer, the hairpin probe-based rolling circle amplification (HP-RCA) technique was developed to directly and accurately distinguish the expression of serum miR-486-5p in NSCLC patients from healthy persons, holding a great potential for further applications in the clinical diagnosis of lung cancers^28^. Moreover, miRNA-30a was significantly elevated in plasma of NSCLC patients. ROC curve analysis showed that the cut-off points of specificity and sensitivity of NSCLC were 61.0% and 84.3%, respectively^29^. Generally, targeted therapy for NSCLC requires more accurate tumor classification. It was found that hsa-miR-205 and hsa-miR-21 expressions were appropriate measures allowing a sufficiently accurate discrimination between lung adenocarcinoma (AC) and squamous cell lung carcinoma (SCC) histotype if they were normalized by U6 snRNA and hsa-miR-103^30^. Other circulating miRNAs could also be used as biomarkers for the diagnosis of colorectal cancer^31,32^, chronic cardiovascular diseases^33^ and pulmonary tuberculosis^34^. In present study, we aimed to verify the expression of miR-33a-5p and miR-128-3p in whole blood and found that the expression levels of miR-33a-5p and miR-128-3p in whole blood of lung cancer patients were significantly lower than those of healthy controls. Compared with plasma and serum, it is more convenient to get whole blood from patients. Also, the stability and abundance of RNA in whole blood is much better than that in plasma or serum. The advantages of minimally invasive and real-time detection of whole blood bring great convenience to clinical diagnosis.

Studies have shown that combined biomarkers can improve the diagnostic accuracy of cancer^35–37^. In the results, the diagnostic values of miR-33a-5p and miR-128-3p in whole blood of lung cancer patients and that of healthy controls showed that the AUC of miR-33a-5p was 0.870, the AUC of miR-128-3p was 0.9278, and the combined AUC of miR-33a-5p and miR-128-3p was 0.951, suggesting a higher combined diagnostic value of miR-33a-5p and miR-128-3p. In addition, the combined sensitivity and specificity of miR-33a-5p and miR-128-3p are better than that of miR-33a-5p and miR-128-3p alone. Compared with a three-miRNA signature (miR-125a-5p, miR-25, and miR-126) as a combined diagnosis of lung cancer^12^, the values of AUC and sensitivity of the combined diagnosis of our two miRNAs (miR-33a-5p and miR-128-3p) are much higher (AUC, 0.951 vs. 0.936; sensitivity, 96.70% vs. 87.50%), but the combined specificity is slightly lower (83.30% vs. 87.5%). We also compared the diagnostic values of miR-33a-5p and miR-128-3p with traditional diagnostic tumor markers, and found that the diagnostic value of miR-33a-5p and miR-128-3p was higher than that of traditional tumor markers. We analyzed the relationship between the expression of miR-33a-5p and miR-128-3p in lung cancer tissues and clinicopathological factors found that both miR-33a-5p and miR-128-3p were related to TNM staging, and miR-128-3p is also related to smoking and tumor size, suggesting certain mechanism in the future research. Notably, the stability of miR-33a-5p and miR-128-3p in whole blood demonstrates an advantage as a joint biomarker for early diagnosis of lung cancer. Since miR-33a-5p and miR-128-3p are stable in whole blood under different harsh conditions, it is speculated that these two miRNAs may be secreted in exosomes, which requires us to verify in future work. Because the cargo of the exosomes are specific to the parental cells and the conditions in which they are produced, suggesting that circulating miRNAs in the exosomes are likely to be prognostic and predictive biomarkers^38,39^. As diagnostic tumor markers, higher expressions of plasma exosomal miR-1290 and miR-375 were significantly associated with poor overall survival for castration-resistant prostate cancer (CPRC) patients^40^. Additionally, circulating exosomal miR-425-3p in NSCLC patients could serve as a biomarker for predicating the clinical responses to platinum-based chemotherapy^41^.

To clarify the diagnostic values of miR-33a-5p and miR-128-3p in lung cancer, we should also expand the sample number to verify its value in future study. To avoid false positive rate, we should also distinguish the blood miR-33a-5p and miR-128-3p in other benign lung diseases (such as pneumonia, tuberculosis, etc) and their expressions in lung cancer. In addition, we tested serum samples from 27 lung cancer patients who received chemoradiotherapy and found that the expression levels of miR-33a-5p and miR-128-3p from lung cancer patients receiving chemoradiotherapy were much higher than that of whole blood with lung cancer, and was lower than that of healthy serum controls (data not shown). We speculate that miR-33a-5p and miR-128-3p are also related to radiotherapy and chemotherapy, suggesting that a detailed investigation is needed.

In summary, both miR-33a-5p and miR-128-3p has the advantage for diagnosing lung cancer at early stage, and the combined diagnosis value of miR-33a-5p and miR-128-3p is much higher. In addition, the diagnostic values of miR-33a-5p and miR-128-3p are superior to traditional tumor markers. More importantly, the expression of miR-33a-5p and miR-128-3p in whole blood is very stable under different harsh conditions, indicating that the two-miRNA signature (miR-33a-5p and miR-128-3p0 has the potential to serve as novel biomarker to distinguish early-stage lung cancer from healthy controls.

## Methods

### Clinical specimens

Clinical samples of lung cancer tissues and paired normal tissues were collected from the Affiliated Hospital of Medical School of Ningbo University (Ningbo, China) and Ningbo Medical Center Lihuili Eastern Hospital (Ningbo, China), and the whole blood samples were obtained from the Affiliated Hospital of Medical School of Ningbo University (Ningbo, China) between September 2014 and December 2017. A total of 65 patients with primary lung cancer and their corresponding normal tissues were resected from the operation before treatment. And the adjacent normal tissues were at least 5 cm away from lung cancer tissues. All tissues specimens were placed immediately after resection in the RNAstore (CWBIO, Beijing, China), then stored at −80 °C until use. Peripheral whole blood of 90 patients with lung cancer and 90 healthy controls were obtained using ethylenediaminetetraacetic acid (EDTA) tubes before surgery. Whole blood samples were placed at −80 °C. All specimens were confirmed to be lung cancer according to the International Cancer Control Alliance Version 8 lung cancer standard system. Written informed consent was obtained from all patients and the study protocol was approved by the Clinical Research Ethics Committee of Medical School of Ningbo University. All experiments were performed in accordance with relevant guidelines and regulations.

### RNA extraction

Total RNA including miRNA, was isolated from tissue samples using TRIzol reagent (Invitrogen, Carlsbad, CA, USA) according to the manufacturer’s instructions. Total RNA containing miRNA in whole blood was extracted by TRIpure LS Reagent (BioTeKe, Beijing, China). First, added 750 ul of TRIpure LS Reagent to the whole blood of 250 μl of lung cancer patients, mixd by vortexing and placed on ice for 10 min. Thereafter, 200 ul of chloroform was added, mixed by vortexing and placed on ice for 3 min. Subsequently, it was centrifuged at a speed of 12,000 g for 15 min. After centrifugation, the supernatant was pipetted into a clean RNase-free eppendorf (EP) tube, 500 ul of isopropanol was added, gently mixed upside down and placed on ice for 10 min. Then, it was centrifuged at a speed of 12,000 g for 10 min. The liquid in the EP tube was poured out, and 1 ml of 75% ethanol in Diethy pyrocarbonate (DEPC) water was added, and the mixture was centrifuged at 8000 g for 5 min, and the operation was repeated once. Finally, the liquid in the EP tube was poured out, the EP tube was placed in air dry, and then the RNA was dissolved with an appropriate amount of DEPC water. Subsequently, the concentration and purity of RNA were measured by DeNovix DS-11 Spectrophotometer (DeNovix, DE, USA).

### Reverse transcription

Using the Hairpin-it^TM^ miR qPCR quantification kit (GenePharma, China). Each reverse transcription was carried out in a volume of 20 μl containing 4 μl 5 × MMLV RT Buffer, 0.75 μl dNTP (10 mM), 1.2 μl miRNA&U6 snRNA RT primer mix (1 μM), 0.25 μl RNasin (40U/μl), 0.2 μl MMLV Reverse Transcriptase, 1 μg total RNA). The reaction mixture was incubated at 25 °C for 30 min, 42 °C for 30 min and 85 °C for 5 min on a Thermal Cycler (MJ research, USA).

### Quantitative real-time PCR

To quantitate miR-33a-5p and miR-128-3p levels, the Hairpin-it^TM^ miR qPCR quantification kit (GenePharma, China) was used for the quantitative real-time polymerase chain reaction (qRT-PCR), and the qRT-PCR reaction was carried out on a Mx3005P real-time PCR System (Stratagene, CA, USA). The qRT-PCR reaction volume is 20 μl, including 10 μl of 2 × Real-time PCR Buffer 1, 0.4 μl of miRNA specific primers (0.1 μM), 2 μl of miRNA RT product, 0.2 μl of Taq DNA polymerase (5U/μl) and 7.4 μl of double distilled water. The reaction was performed in an eight-strip (Axygen, USA) tube under the following conditions: 95 °C for 3 min, 40 cycles of 95 °C for 12 s, 62 °C for 40 s. The small nuclear RNA U6 was used to normalize the expression of miR-33a-5p and miR-128-3p. The relative expression levels of miR-33a-5p and miR-128-3p were calculated using the 2^−∆CT^ method All qRT-PCR reactions were performed in triplicate.

### Cell culture

Four lung cancer cell lines (NCI-H1299, A549, SPC-A-1, LTEP-a-2), and the human normal lung epithelial cell line (BEAS-2B) were purchased from Chinese Academy of Sciences Cell Bank of Type Culture Collection (Shanghai, China). NCI-H1299, A549, SPC-A-1 and LTEP-a-2 cell lines were cultured in RPMI-1640 (Gibco, USA) medium supplemented with 10% fetal bovine serum (PAN-Biotech, Aidenbach, Germany) with 1% penicillin/streptomycin (Life Technologies, CA, USA). BEAS-2B was cultured in Dulbecco’s modified Eagle’s medium (DMEM) high glucose (HyClone, CT, USA), containing 10% fetal bovine serum only. All of these cells were maintained in 5% CO_2_ at 37 °C.

### Statistical analysis

All data were analyzed by using Statistical Product and Service Solutions software 23.0 (Statistical Product and Service Solutions, IL, USA) and GraphPad Prism 7.0 (GraphPad Software, CA, USA). The Wilcoxon matched-pairs signed rank test was used to compare the expression levels of miR-33a-5p and miR-128-3p between lung tumor tissues and paired adjacent normal tissues. The Student’s t-test was performed to compare the different expression levels of miR-33a-5p and miR-128-3p between lung cancer cell lines and human normal lung epithelial cell line. The Nonparametric Mann-Whitney U test was employed to compare the expression levels of miR-33a-5p and miR-128-3p in whole blood between lung cancer patients and healthy person. The Chi squared test was used to analyze the associations between the expression level of miR-33a-5p and miR-128-3p and the clinicopathological factors. The diagnostic value of miR-33a-5p and miR-128-3p were conducted by the ROC and the AUC. The optimal cut-off thresholds were determined by using the highest Youden index. For correlation, Pearson’s correlations were used. For all analyses, differences were considered statistically significant at *P*-value less than 0.05.

## Electronic supplementary material

Supplementary Information
