# Supplementary material for: A two-miRNA signature (miR-33a-5p and miR-128-3p) in whole blood as potential biomarker for early diagnosis of lung cancer
Source: Sci Rep. 2018 Nov 12;8:16699. doi: 10.1038/s41598-018-35139-3 (PMC6232109; doi:10.1038/s41598-018-35139-3)
Supplement: Supplementary file 1 — Supplementary Information [file 41598_2018_35139_MOESM1_ESM.pdf]

# A two-miRNA signature (miR-33a-5p and miR-128-3p) in whole blood as potential biomarker for early diagnosis of lung cancer

Jinchang Pan[1,2,‡], Chengwei Zhou[3,‡], Xiaodong Zhao[3], Jinxian He[4], Hui Tian[4], Weiyu Shen[4],Ying Han[5], Jun Chen[5], Shuai Fang[1,2], Xiaodan Meng[1,2], Xiaofeng Jin[1,2], Zhaohui Gong[1,2*]

## Supplemental Materials

**Supplementary Fig. S1. The specificity of qRT-PCR products for miR-33a-5p and miR-128-3p.** The dissociation curves of miR-33a-5p (**A**) and miR-128-3p (**B**) respectively showed that the amplified products yielded a single peak.

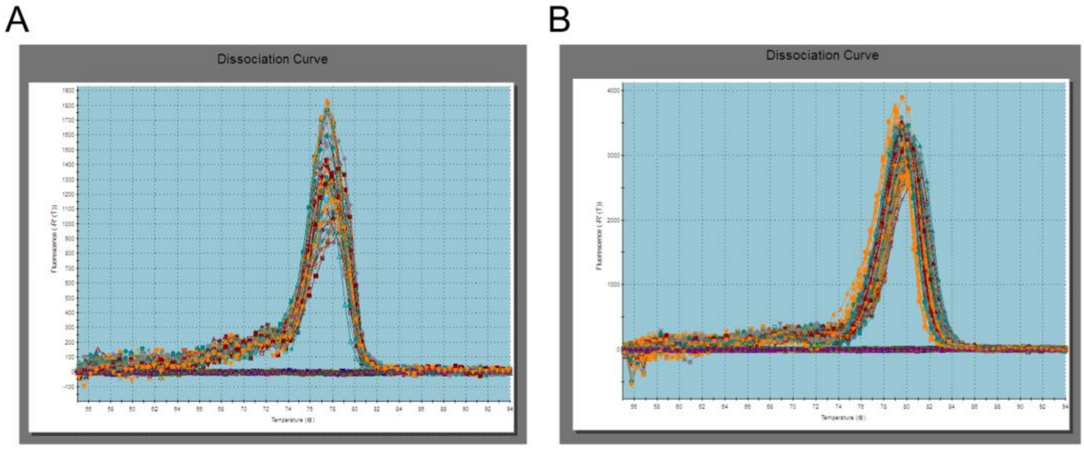


**Fig. S1**
